# Defining Transition and Transition Success: Perspectives From Different Stakeholders

**DOI:** 10.1111/jar.70199

**Published:** 2026-03-04

**Authors:** Jan Šiška, Julie Beadle‐Brown, Šárka Káňová, Tereza Havránková, Marie Černíková

**Affiliations:** ^1^ Faculty of Education University of West Bohemia in Pilsen Pilsen Czech Republic; ^2^ Faculty of Education Charles University Prague Czech Republic; ^3^ University of Kent Canterbury UK

**Keywords:** intellectual and developmental disability, support, transition, young adults

## Abstract

**Background:**

Although research has identified that the transition to adulthood for young persons with intellectual and developmental disabilities is not linear, little is known about how young persons with intellectual and developmental disabilities, parents, and teachers conceptualise transition, particularly successful transition. The focus of this paper is on how the different stakeholders conceptualised transition and what constitutes successful transition, in general, and for persons with intellectual and developmental disabilities.

**Method:**

Thematic analysis was applied to 8 focus groups with 84 participants, exploring transition and successful transition in the Czech Republic.

**Results:**

Five themes emerged, including gaining independence, having a job and opportunities as everybody else, satisfaction with life, and transition of youth with intellectual and developmental disabilities as an important issue which needs to be addressed.

**Conclusions:**

Our findings point to the need for recognising different outcomes of successful transition from postsecondary education to adulthood, not just those primarily related to employment.

## Lay Summary

- Participants understood transition in terms of outcomes. Successful transition is about gaining independence, which comes with increased responsibility. Having a job and inclusion of other forms of meaningful activities is also important for a successful transition. This is important in a global climate of economic crisis, increasing technology and automation, and fewer jobs for the general population.
- The importance of transition for youth with intellectual and developmental disabilities is not often recognised. It needs to be understood and addressed holistically.
- Availability of just the right and diverse support is the key to a successful transition.
- Future research could gather more detailed examples of transition successes and of what happens when transition support is not available or fails.

## 1 Conceptualising Transition to Adult Life and What Constitutes Successful Transition

Transition to adulthood for young persons with intellectual and developmental disabilities has been highlighted in international literature as being of particular importance to the outcomes experienced as adults (Chun et al. 2022; Morris 2002). Notwithstanding the United Nations Convention for the Rights of Persons with Disabilities (United Nations 2006), young people with intellectual and developmental disabilities and complex needs often transition from special education provisions to other congregated settings including care homes or sheltered employment due to a shortage of suitable alternatives (Ormston et al. 2017; Šiška et al. 2018).

Preliminary findings from a scoping review currently underway by the research team suggest that, although only approximately one‐fifth of studies offered a definition or detailed description of transition (as a concept or process), the notion of transition as being a period of “emerging adulthood” for students with and without disabilities is a common theme in the literature (anonymised). The concept of “emerging adulthood” is discussed both in the context of a chronological change from adolescence to adulthood as well as a period of youth development and a change from being a student in school to assuming adult roles and responsibilities. Similarly, a common discussion in the literature identified by the scoping review above focuses on preparation for life changes as a component of transition. Such changes include employment status, education status, daily living and routines, community living, health and medical, and relationships.

Like the concept of transition more generally, only 21% of the studies included in the scoping review provided a definition or description of a successful transition. Of the studies that discussed successful transition, a range of different aspects of success was discussed, from a range of different sources and countries. The most common focus was on employment as an indicator of successful transition, followed by independent living, and post‐secondary education.

However, such indicators of a “successful” transition have been considered by Pearson et al. (2021) as normative or even potentially damaging. Transition from mainstream school to adult life is an unpredictable and difficult period for all students as well as for their families/caregivers. However, this period is particularly difficult for those with disabilities who often face additional barriers to achieving these indicators (Beadle‐Brown et al. 2023). For many people with disabilities, support is required through both the process of transition, typically to employment or independent living, and into adulthood itself. The higher people's support needs are, the more likely they are to access an increasing and varied range of services, which need to work together to ensure a successful transition. Regrettably, research suggests that often services are not organised holistically (in terms of thinking about all elements of the young person's life or across the life span), and that often there is a gap (sometimes even a “cliff” or “black hole”) between child and adult services and environments (Biswas et al. 2016). Lack of coordination between different agencies involved in the lives of people with disabilities has also been identified as a key barrier to positive outcomes, including the development of community living and participation as active citizens in society (Šiška and Beadle‐Brown 2022).

This paper reports on research conducted in the Czech Republic. Education for persons with intellectual disabilities has a long tradition in the Czech Republic, going back to the nineteenth century when the first special schools were established. The first Czech special educators promoted not only formal education for persons with intellectual disabilities but also providing them with support during transition and after leaving school or a residential institution (Černá et al. 2015). As in other European countries, people with intellectual and developmental disabilities in the Czech Republic have traditionally been educated in special schools and institutions. The Czech Republic has ratified the United Nations Convention for the Rights of Persons with Disabilities (UN CRPD 2006), which requires state parties to construct education systems that provide the support necessary for community living and active participation. Many EU member states have moved in the direction of inclusive education, which has led to increasing numbers of learners with disabilities in mainstream education (European Agency for Special Needs and Inclusive Education 2018). However, the proportion of children with intellectual disability in mainstream education is considerably lower than that of those with other forms of special educational needs (SEN) (Buchner et al. 2020). Czech research on transition focuses largely on transition from school to employment (Trhlíková 2020) with little attention given to other elements of transition and without any discussion of what constitutes successful transition to employment.

This is reflective of the international literature more generally in which the majority of studies refer to employment (e.g., Chun et al. 2023; Lucas et al. 2022) as an important outcome of successful transition. The next most commonly identified outcome is independent living (e.g., Pillay et al. 2022), followed by post‐secondary education (e.g., Pillay et al. 2020; Skillern and Carter 2021). Flannery and Hellemn (2015) described the ultimate outcome of school as “success in adulthood: stable and competitive employment, independent living, and satisfaction with one's quality of life” (p. 67).

There is a relatively substantial amount of research on models of successful transition to adulthood for young people without disabilities (e.g., Birkeland et al. 2012; Brown et al. 2005) but less research related to predictors of transition success for individuals with intellectual disability. Bronte one‐the‐less, some key factors have been identified—For example, acquiring skills, such as academic, employment, self‐awareness, and self‐care skills (Lee et al. 2019), having skills in self‐advocacy and having self‐determination (e.g., Grenwelge and Zhang 2013; Nadig et al. 2018), developing relationships (e.g., Young et al. 2020), as well as community inclusion (e.g., Francis et al. 2018; Miller et al. 2018).

## 2 Purpose of the Study and Research Questions

Very little of the research to date has directly involved young people with or without disabilities themselves in the construction of definitions or concepts related to transition. The current paper seeks to bridge this gap and reports findings from a qualitative exploration of the views of a range of different stakeholders in the Czech Republic, including young people with and without disabilities and young people currently preparing to leave school, as well as those who have already made the transition from school to adult life.

The Czech Republic is not so different from many other countries in Europe and indeed across the world, particularly those that were impacted by a socialist regime and where transition to community‐based started much more recently (Šiška and Beadle‐Brown 2021). The UN CRPD provides the current backdrop for service provision in the majority of these projects although implementation is patchy in most of these countries (Šiška and Beadle‐Brown 2021). The educational system is similar to that in other European countries—despite much discussion on inclusive education, there remains a substantial system of special schools for young people with intellectual and developmental disabilities and segregated congregate settings for adults, especially for those with higher support needs. As such, this study provides the opportunity to explore topics that have relevance on an international level.

## 3 Research Questions

This study is part of a broader project developing our understanding of how educational and social care systems can support young people with intellectual and developmental disabilities to transition successfully from school or other forms of education to adult life. The current study focused on answering two core research questions drawing on a wider range of stakeholder views than has been the case in previous research:

Research question 1 How is transition to adulthood conceptualised by young people, parents and teachers in the Czech Republic and do views differ by stakeholder group.

Research question 2 What are the characteristics and elements that indicate successful transition to adulthood?

## 4 Methods

### 4.1 Design

This qualitative study used focus groups with a range of different stakeholders, each bringing a different perspective on the topic of transition. Focus groups were used as they permitted participants to communicate their experiences, perceptions, relate opinions, and generate ideas grounded in each other's insight (Krueger and Casey 2014).

All procedures performed in this study were in accordance with the ethical standards of the institutional research committee and with the 1964 Helsinki Declaration and its later amendments. Informed consent was obtained from all individual participants involved in the study.

### 4.2 Participants and Recruitment

The aim was to understand transition through the perspectives of diverse individuals who were directly engaged in transition, including persons with intellectual and developmental disabilities, parents, educators, and other professionals. By employing focus groups with a diverse range of stakeholders, the study achieves a commendable depth and breadth of insights.

Participants were initially identified and contacted through the research team's networks and professional connections. All participants were recruited from two regions in the Czech Republic, one in the North of the country and one in the West. There are no notable socioeconomic variations between the regions. Both regions comprised a combination of small urban areas and mid‐sized cities. Purposeful sampling was applied to ensure theoretically relevant stakeholder groups (Maxwell 2013). Eight focus groups were formed intentionally—two groups conducted for each group of stakeholders (see below), one group in each region. The number of participants in each focus group ranged between 3 and 8 as suggested by Krueger and Casey (2014). Eighty‐four participants in total were recruited. Table 1 illustrates the gender and age range for each of the participant groups.

**TABLE 1:** Gender and age of respondents in each focus group.

Respondent category	Number of respondents	Male	Female	Age
Special school teachers	9	1	8	—
Regular school teachers	10	1	9	—
Parents of graduates with intellectual and developmental disabilities	10	1	9	41–58
Graduates with intellectual and developmental disabilities	13	7	6	19–26
Other professionals	11	4	7	—
Graduates without intellectual and developmental disabilities	8	2	6	20–25
Students without intellectual and developmental disabilities	13	2	11	18–19
Students with intellectual and developmental disabilities	10	6	4	18–26

*Teachers from special schools* (*n* = 9) were recruited via professional networks. To be included, teachers from special schools needed to have at least 5 years of professional experience in educating adolescents with intellectual and developmental disabilities.

*Teachers from mainstream schools* (*n* = 10) were all recruited via professional networks of schools. Inclusion criteria for teachers comprised having at least 5 years of teaching experience with adolescents and being actively engaged in supporting students in transition‐relevant programmes.

*Parents of young persons with intellectual and developmental disabilities* (*n* = 10) were all recruited via professional contacts of schools and family support groups. Parents were included if they were the primary caregivers of a young person with intellectual and developmental disabilities who was currently in the final year of school and thus experiencing transition, or who had already transitioned from school no more than 6 years previously.

*Other professionals* (*n* = 11) were all recruited via professional connections of the research team. Professionals were included if they had at least 3 years of experience providing a variety of service types of transition‐related services (e.g., social services, employment) for persons with disabilities and their families.

*Young people with intellectual and developmental disabilities—students* (*n* = 10) were all recruited via professional networks of schools that educated youth with intellectual and developmental disabilities. The students were recruited from special schools which educate predominantly students with mild or moderate intellectual disability. The inclusion criteria comprised having a primary diagnosis of intellectual disability and being between 18 and 26 years of age. No formal assessment of cognitive or adaptive functioning was conducted. But all had at least some communication difficulties and focus group discussions were facilitated using plain language and visual aids such as pictures.

*Young people with intellectual and developmental disabilities who had already left school* (*n* = 13) were all recruited via the networks of their previous schools. Inclusion criteria were a primary diagnosis of intellectual disability; being aged between 19 and 26; and having graduated from school at least three and no more than 6 years previously.

*Young people without intellectual and developmental disabilities—students* (*n* = 13) were all recruited via schools in the two regions. The inclusion criteria comprised being between 18 and 19 years old and being in the final year of high school education.

*Young people without intellectual and developmental disabilities who had already left school* (*n* = 8) were all recruited via the network of undergraduate students of the university, which implemented this research project. The inclusion criteria involved being aged between 19 and 26 years and having graduated from high school education.

### 4.3 Materials and Procedures

The focus groups took place in rooms or classrooms of the schools for young people with intellectual and developmental disabilities, for parents of young people with intellectual and developmental disabilities, online for teachers, for other professionals, and for students and graduates without intellectual and developmental disabilities. To avoid the potential discomfort of youth with intellectual and developmental disabilities in responding to some of the questions, only researchers were present in the classrooms. As the participants entered the room, they were reminded about the purpose of the meetings and asked to sign the consent forms. The consent form for people with intellectual and developmental disabilities was prepared in an easy‐to‐read format, and the content was also verbally explained. All moderators were experienced in conducting qualitative research. The two moderators who led the focus groups with young persons with intellectual and developmental disabilities and their parents had experience in intellectual and developmental disabilities, postsecondary transition, and group counselling. For most focus groups, there were two moderators present—a lead moderator and a second moderator primarily responsible for administration and recordings. The conversations in the focus groups were audio‐recorded, transcribed verbatim using transcription software, and checked for completeness and clarity by the second moderator. Participants were not compensated for their contribution.

### 4.4 Focus Group Schedule

The schedule used to guide the focus groups was developed using Krueger and Casey's (2014) guidelines on focus groups. In addition, a set of semi‐structured, open‐ended discussion questions were constructed and shared via PowerPoint presentation to encourage participation. For each open‐ended question, a series of more detailed prompts was also presented by PowerPoint. For participants with intellectual and developmental disabilities, the interview protocols were developed in an easy‐to‐read format accompanied by pictograms to enable better comprehension of the questions discussed. The same schedule and protocol were used with all eight groups, except for how the target audience was referred to (e.g., “your students,” “your son/daughter”; “you” for young persons with intellectual and developmental disabilities and other students). Topics explored in the focus groups included:

1. How is a successful transition to adulthood defined and conceptualised in different contexts and by different stakeholders?
2. What are young people's experiences of transition, including transition outcomes?
3. What are the current approaches to supporting young people with intellectual and developmental disabilities through the transition from school to adult life?
4. What are the factors or approaches that facilitate or impede successful transitions, and develop a logic model to inform future research on this topic?

This paper focuses in particular on Topic 1: How is transition defined? How is a successful transition defined?

- In general
- For individuals with intellectual disabilities, more specifically

## 5 Data Processing and Analysis

Our study was led by the research questions using a top‐down or theoretical thematic analysis. Braun and Clarke (2006) define a “theme” as a pattern that captures something significant or interesting about the research question. The coding team consisted of four members. One member with broad experience in qualitative and thematic analyses developed the coding process.

Analysis was guided by the six‐step process outlined by Braun and Clarke (2006, 18): Step 1: Become familiar with the data, Step 2: Generate initial codes, Step 3: Search for themes, Step 4: Review themes, Step 5: Define themes, Step 6: Write‐up.

During the first stage of the open coding, two members of the coding team were assigned as the primary and secondary coders for each focus group in both regions. Both read the entire individual transcript and designated particular quotations and independently recorded analytic memos using MAXQDA. The two coders then met to discuss and agree on the codes. The initial codebook was then constructed using the quote inventories from the primary and secondary coders (following Glaser and Strauss 1967). And then the whole team met to review the code book, and each code was then refined or expanded to better capture different connotations of the codes until agreement was reached.

When coding was complete for all 16 focus groups (8 in each region), the coding pairs began the process of organising the codes into themes and, where relevant, sub‐themes. As for the coding phase, initial theme identification was conducted independently by the two researchers, and then they met and discussed their respective themes until consensus on the final set of themes was reached. During the development of the themes, the coders also produced analytical memos regarding similarities, differences and relationships using MAXQDA data management and analysis software.

## 6 Reflectivity and Data Reliability

The research team brought together people from different disciplines and with different perspectives. All but one researcher already held a Ph.D. in either special education, education, anthropology, or art education, with the remaining researcher in the process of completing their doctoral programme. Two members of the research team (including the Lead researcher) had experience directly supporting individuals with intellectual and developmental disabilities, parents, and staff in social care services and the education system in the Czech Republic and abroad, and both work to inform and support improvement in the nature and quality of support and quality of life of people with disabilities. Their knowledge of the education and social service system was primarily an advantage in that they were very familiar with the context in which the focus groups were being conducted. However, this could also result in some bias and the potential for them to make assumptions about what people were referring to and not ask individuals to explain their responses. To enhance transparency and a collaborator who was not familiar with the Czech context was invited to evaluate the coding framework and thematic analysis for objectivity.

Several reliability and validity strategies, including peer debriefing, reflexivity, and triangulation (Lub 2015), were used to (1) recognise and minimise bias, (2) ensure that potentially important but different perspectives were included, and (3) ensure the reliability of the findings.

Firstly, all researchers were involved at least to some extent in all phases of the research, including the development of initial codes generated in the analysis. Regular team meetings were held to discuss guidance on the research methods and analysis process for the focus groups, to review coding systems and initial analysis, and to debate researchers' positions and perspectives at different stages in the data collection and analysis process.

Secondly, as noted above, initial open coding of the data from each focus group was conducted by two researchers, initially independently and then they met to discuss and agree on the coding structure as described above. Following this, the whole team met to bring together the coding book before focus coding was undertaken, again by members of the coding team working in pairs. Researchers reflected freely during all of these meetings about their different perspectives and potential biases in the coding.

As also noted above, theme identification was led by one researcher, but with a second researcher also independently identifying themes for all focus groups. Consensus on themes and subthemes was reached through discussion.

## 7 Findings

**FIGURE 1:**
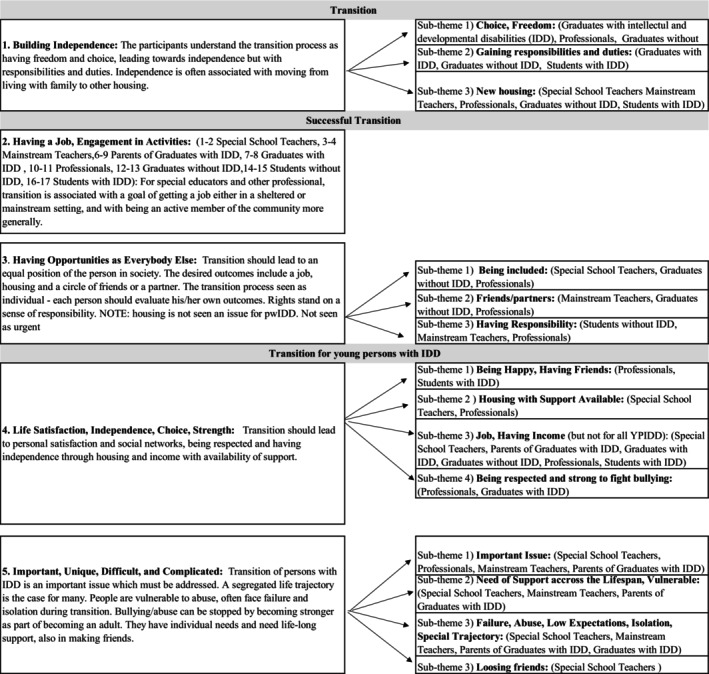
Diagrammatic representation of themes, subthemes, and definitions.

In this paper, we have concentrated on the data that specifically addressed how transition and successful transition to adulthood are understood and defined in diverse contexts and by different participants. Figure 1 summarises the themes, subthemes, and key definitions.

As can be seen from Figure 1, the analysis generated five themes across all participant groups. Table 2 below illustrates from which participant groups each theme was generated.

**TABLE 2:** Mapping which of the five themes were identified by which groups.

Theme	Teachers—special school	Teachers mainstream	Parents of graduates with IDD	Students with IDD	Graduates with IDD	Students without IDD	Graduates without IDD	Professionals
1. Transition is about: *Building Independence*	✓	✓	✓	✓	✓	✓	✓	✓
2. Transition is about: *Having job, Engagement in Activities*	✓	✓	✓	✓	✓	✓	✓	✓
3. Successful transition is about: *Having opportunities similar to everyone else*	✓	✓					✓	✓

Theme	Teachers—special school	Teachers mainstream	Parents of graduates with IDD	Students with IDD	Graduates with IDD	Students without IDD	Graduates without IDD	Professionals
1. Transition for young people with IDD—*Social Networks, Independence, Choice, Strength*	✓		✓	✓	✓		✓	✓
2. Transition for young people with IDD is *Important, Unique, Difficult, and Complicated*	✓	✓	✓		✓			✓

Two themes (Theme 1 and 2) emerged from the discussion focusing on transition in general, and one centred on what is a “successful” transition (Theme 3). Themes 4 and 5 were generated by the questions asking people to think about transition for people with intellectual and developmental disabilities more specifically. However, it is important to note that participants found it hard to distinguish between the definition of “transition” and that of “successful transition” so there are some overlaps between these themes. In addition, there were both similar sub‐themes across those with and without intellectual disabilities and unique sub‐themes; and sometimes, when asked about transition more generally, individuals answered specifically in relation to people with developmental disabilities.

### 7.1 Conceptualising Transition

#### 7.1.1 Theme 1: Building Independence: “It's About Taking Over Responsibility for One's Own Life”

Although this theme emerged in one way or another from all focus groups, there were both similarities and differences in how each group formulated this theme. All groups, apart from the mainstream and special schoolteachers, conveyed an overall sense of transition being about *Building Independence*, particularly in terms of the process of gaining freedom and having options to choose from. They further elaborated that building independence is closely related to having *choice and freedom* in forming part of the process of transition to adulthood.

Young people, as they become adults, become independent. This means that they live and work alone, take care of their affairs, have satisfactory family and partner relationships, and are able to simply take responsibility for their own life.

Young people themselves, from all three groups, particularly noted the intersection between independence and *gaining responsibilities and duties*. One young person without intellectual and developmental disabilities commented that transition was a process of fading dependence on parents:

I am simply now organising my life without parents' help.

Under this theme, almost all groups discussed making decisions that contribute to personal growth, empowerment, and acquiring essential life skills needed for having responsibility, pursuing educational and vocational paths, and a self‐sufficient lifestyle, housing included. The following example from one student with intellectual and developmental disabilities particularly addresses gaining responsibility for one's own life:

I think that maturity does not have to be related to age at all. That he has to take care of himself. He has to realize that he is no longer the center of attention, and he has to look after himself and face situations that he has not yet experienced.

Both groups of teachers, professionals, and graduates without intellectual and developmental disabilities, and students with intellectual and developmental disabilities stated that transition towards independence relates to *new housing* arrangements other than with living with the family. One student with intellectual and developmental disabilities mentioned the connection between transition to adulthood and moving from the family.

So, as soon as a person becomes an adult it is possible to move out.

In summary, this theme focused on transition as the process of growing in independence and self‐determination. All groups of students and graduates—those with and without intellectual and developmental disabilities—discussed not only increased freedom and independence as part of the transition to adulthood, but also accepting responsibilities and duties. This was often conceptualised as moving out of the family home and taking increasing responsibility for themselves. However, parents of young people with intellectual and developmental disabilities did not refer to the young person moving out of the family home and living more independently.

#### 7.1.2 Theme 2: Having a Job; Engaging in Activities: “Employment is the Completion of Transition”

Special schoolteachers and professionals emphasised that *having a job* is a desirable outcome.

Employment! Because from my point of view, it is the culmination of the whole growing up. Just the moment that person starts working, taking care of himself, and solving these common problems, that's how I think he's matured.

Young people with intellectual and developmental disabilities also mentioned the importance of work:

Work is important – find work because work will feed you. And you simply have the money to pay the mortgage so you can enjoy the apartment. That's great then.

However, the participants did not refer to transition as leading only to paid employment.

Transition support should navigate a person to be engaged in various meaningful activities in community and family.

A special teacher defined transition as a series of domains in life that relate to all people regardless of ability or disability:

It doesn't matter if he has a disability or not. Basically, there are four pillars: work, family, hobbies, and friends. We all have it. It should be something like a fan. This means, simply, that in adulthood each of us should have a job, hobbies, friends, and family.

Although this part of the discussion focused not just on the transition from the perspective of people with intellectual and developmental disabilities, parents discussed their perspective on successful transition, particularly referring to their children with intellectual and developmental disabilities. Parents were appreciative of their children with intellectual and developmental disabilities being *engaged in activities*. One parent shared his or her experience of a successful transition:

She goes shopping with some help. She cooks and bakes. From this perspective, I am glad.

One teacher from a special school shared his perspective of transition as leading to community participation. However, he referred to community participation for those with mild intellectual disabilities and with family support.

In summary, engagement in employment was seen as a catalyst for promoting independence through income. However, paid work was not seen as the only goal. Opportunities for contributing to overall well‐being and community participation were also highlighted.

### 7.2 Successful Transition

#### 7.2.1 Theme 3: Having Opportunities as Everybody Else: “It is About Being Active, Really”

All groups, except parents, emphasised being included and *having the same opportunities* for inclusion, choice, responsibility, and relationships as other members of society.

*Being included* was particularly referred to as having a life similar to others, participating, and being active as an individual, as illustrated by one of the participants in the Professional focus group.

It is about being active, really. About making progress as a person.

Teachers from special schools associated successful transition with social inclusion more generally. One special teacher highlighted that:

Well, I think, success is when the individual is included. It is a goal for any young person.

Mainstream teachers, graduates without disability, and professionals pointed out that building a new *network of friends* was an important element of successful transition and key to happiness. This important relationship was also echoed by graduates with intellectual and developmental disabilities who expressed their uncertainties about losing their friends from school after graduation.

Although I was really pleased that I completed school, I was sad that I left my friends.

When we finish school, we lose friends that we have spent many years with.

As noted in one of the earlier sections, they also highlighted that a successful transition involved *duties and responsibilities* as well as having rights.

My perspective is that a person takes responsibility for him or herself, pays the rent, shopping, etc.

Young people without disabilities—both students and graduates—and professionals concluded that a universal definition of successful transition cannot be articulated since success in transition depends on an individual interpretation of success. Only the individual can say what success in transition is and what this person would like to see as outcomes of transition, as stated by this professional:

It depends on how the person perceives himself, what success is for them. Because we all have our bar set at different levels.

Although this part of the discussion focused on successful transition more generally, teachers from special schools and professionals tended to specifically relate the topic to young people with intellectual and developmental disabilities. The transition from school to employment without a transition gap was commented on as a potentially significant achievement. One teacher from a special college shared:

It's great if they keep them straight after school and at a specific workplace where they had their vocational practice. If they immediately offer them a job there, that's a big, big success. For example, we were finishing up nursing services at the girls' school and were just about to start working at a nursing home. The girls were so handy that they hired them there too.

One professional discussed success in the transition of young people with intellectual and developmental disabilities about moving from an institution to a less protected living environment:

I simply consider all our clients to have been successful. Because for us it was a transition from an institutional environment to a less dependent way of life, or as independent as they can manage.

Even though making new friends after leaving school was considered an important element of successful transition more generally, teachers from special schools referred not only to friendship but also to partnership. A successful transition story was shared about a young woman with intellectual and developmental disabilities who, together with her partner, learned to look after their baby during the transition process, despite the difficulties.

We had a girl who got pregnant (during her vocational training, authors' note) and finished that school successfully. The couple was from the same school grade. They were the parents. Both finished school. They were able to learn. He immediately started work, and she took care of the child. Then I would consider it a successful transition. However, to have a child at 18, I think it's very difficult.

In summary, although this theme arose out of the discussion on successful transition more generally, some participants referred specifically to people with intellectual and developmental disabilities in their responses. The parent focus groups did not contribute to this part of the discussion (their views were elicited more in the discussions on people with intellectual and developmental disabilities and the experiences of their family members). The successful transition was conceptualised in terms of young people having the same rights and responsibilities as other adults—working, making decisions for themselves, looking after themselves and others (such as children), being included and generally active, and participating in society. Successful transition, as highlighted under Theme 1, was also about moving into more independent living arrangements. Finally, there was also a very important social dimension to a successful transition—with having a partner and building new social networks to compensate for the loss of school friends being emphasised.

### 7.3 Transition and Successful Transition for Persons With Intellectual and Developmental Disabilities

#### 7.3.1 Theme 4: Independence and Social Networks: “There is Always Fear. But You Have to do Something Not to be Afraid”

This theme arose from the discussion that focused specifically on transition and successful transition for young people with intellectual and developmental disabilities. All but two of the focus groups (mainstream teachers and students without intellectual and developmental disabilities) contributed to this theme. Teachers and students with intellectual and developmental disabilities discussed the importance of *life satisfaction* in general terms and the importance of building and enjoying *friendships* as part of this.

A teacher commented:

Shifting in social relationships is important. Social relationships are motivational and contribute to satisfaction.

As noted under conceptualising transition more generally, students with intellectual and developmental disabilities discussed successful transition as being about having your own house/home and having your own family.

It's about buying a house.

Often when talking about independence, the connection between *housing with available support* was made by special schoolteachers and professionals, but not parents. One participant shared that:

The parents are afraid of residential services. Parents see it as an ultimate possibility. They would make arrangements with social services only when they couldn't cope anymore.

On the other hand, parents expressed their scepticism in terms of the potential independence of their children, admitting their strong mutual reliance.

…even the idea that he should move out, I've been with him for 22 years, and now suddenly, what am I going to do?

Again, almost all groups discussed the importance of opportunities for *engaging in employment* for young people with intellectual and developmental disabilities. Special schoolteachers felt that transition should lead to independence and opportunities to make choices. Income as a facilitator of independence was highlighted by this teacher:

First, it is about being successful in finding a job. Then, having a place to live independently.

However, parents felt that employment is not possible for every person with intellectual and developmental disabilities.

Professionals expressed their unique concerns about being respected as a positive outcome to successful transition for young persons with intellectual and developmental disabilities. When graduates with intellectual and developmental disabilities discussed success in transition, the specific areas that were brought up were *choice, independence, and being strong or courageous to deal with difficulties and new experiences*:

There is always fear. But you have to do something not to be afraid.

One graduate with intellectual and developmental disabilities highlighted their experience with bullying and desire to gain strength to fight back:

Growing up to adulthood means to get stronger, to be able to fight back if I get teased and bullied.

In summary, Theme Four was generated by all groups who saw success in transition for the youth with intellectual and developmental disabilities as having independence in living arrangement achieved through the necessary support, accompanied by friends, and the strength to stand up for themselves and respond to bullying. Only students with intellectual and developmental disabilities and professionals highlighted overall happiness or life satisfaction as a positive outcome of transition.

#### 7.3.2 Theme 5: Unique and Difficult: “Transition Needs to be Addressed. It's Important”

Teachers, other professionals, and parents portrayed transition as an *important issue* that must be addressed, particularly because there are some elements unique to people with ID, there are several barriers, and the experience tends to be difficult and worrying.

The perspective of transition as an important issue was elaborated further by teachers and parents. As mentioned in the previous theme, they highlighted the *vulnerability to bullying* of young people with intellectual and developmental disabilities, and *the need for support across the lifespan, including potential gaps in support*. One teacher indicated that:

…the biggest problem for us is that the parents are worried about what will happen to the children when the parents are no longer here, when the parents get old, and then there are really situations where the child will go when the parents are not here anymore.

Special schoolteachers discussed that the *vulnerability* of young people with intellectual and developmental disabilities in social relationships could lead to social *isolation* and hinder their success in integration into the community.

I think social relationships are important for life. We really don't know much about this topic. The topic of social relations of people with mental disabilities is not much discussed.

The support provided to persons with intellectual and developmental disabilities was commented as typically segregated, often navigating through “a special” trajectory across education and training, congregative living opportunities, and engagement in sheltered employment.

On the other hand, graduates with intellectual and developmental disabilities described their frequent experience of *failure and low expectations* about their potential. For instance, this graduate with intellectual and developmental disabilities shared his experience interacting with an employer:

I wanted to work there. Because I know that I'm quite good at it, that I enjoy it. They didn't take me. I mind that. That they simply do not give the opportunity to show that even if we are slow, the quality is there.

Special schoolteachers discussed that young people with intellectual and developmental disabilities are particularly vulnerable in reducing social networks during the transition from school to other domains.

Once they graduate from school, we still have some sort of overview of them for a while. We try, but then we lose touch. Then it's up to them. They appear lonely.

In summary, teachers, parents, professionals, and graduates with intellectual and developmental disabilities highlighted that transition for young people with intellectual and developmental disabilities is an important topic which needs more attention. Although teachers and parents highlighted the need for support across the lifespan, they were focused primarily on friendship and interpersonal relationships.

## 8 Discussion, Conclusions, and Implications

### 8.1 Summary of Key Findings

This paper aimed to explore how transition and successful transition, specifically, are defined or conceptualised by a range of different stakeholders and in different contexts. Much of the previous research did not offer such a definition. In the current study, transition was primarily conceptualised by all participant groups in terms of outcomes, in particular, what changes in people's lives at this time, with increased independence being a primary focus. Participants generally did not conceptualise transition as a process. Similar to the previous research identified by Beadle‐Brown et al. (2023), and Šiška et al. (2024) and the ongoing scoping review, successful transition was conceptualised in terms of things that changed during the transition from school to adulthood, specifically:

- employment—having a job as a source of income, of occupation, and of inclusion
- living situation—moving out of the family home or institutional setting and into a home in the community
- inclusion in the community and having new relationships

Some new findings also emerged from this study, as did some particularly interesting similarities and differences in how transition/successful transition was conceptualised.

First, the importance of employment seen in previous research was slightly tempered by the inclusion of other forms of activities and of generally being engaged in leisure, volunteering, sport, and exercise. This is particularly important in a global climate of economic crisis, increasing technology and automation and generally fewer jobs for the general population—finding just the right job (with the right support) for individuals with intellectual and developmental disabilities becomes even more challenging. Thinking more creatively about work and occupation is necessary in this context. Secondly, this is important because participating in activities like volunteering, clubs, sports, etc. also helps people to build their experiences, skills and networks that may open the door for employment later. In the wider project, there were examples of young people who had, for example, become children's tennis coaches through their own leisure activities.

Secondly, there was recognition, especially by young people themselves, both those with and without intellectual and developmental disabilities, that becoming an adult was also very much about having increasing responsibility in life—looking after yourself more, looking after your home, and your money, not relying on others as much, especially parents. This was also tied to self‐determination and making your own decisions.

Thirdly, young people with and without intellectual and developmental disabilities appeared to think about the transition in similar ways and conceptualised transition as something similar for all young people. In contrast, parents and special schoolteachers appeared to find it harder to think about transition more generally and focused on those with intellectual and developmental disabilities specifically. They tended to refer to transition as being quite different and more difficult for young persons with intellectual and developmental disabilities. Parents appeared to have more limited views of transition success and did not mention leaving home and living independently or in supported accommodation as an indicator of success. They were also less positive about whether their son or daughter could have a job.

The area where young people with and without intellectual and developmental disabilities differed, and where young people with intellectual and developmental disabilities were closer to, for example, teachers in special schools, was about having to be stronger as an adult, particularly in trying new things and dealing with bullying.

### 8.2 Strengths, Limitations, and Implications for Future Research

This study was exploratory and was the first attempt in the Czech Republic to fill some of the gaps in the existing literature on the transition from school to adulthood and adult life. Having seven different groups of participants representing different perspectives was a strength. Including both those with and without intellectual and developmental disabilities was also a strength in starting to understand how different or unique the experience of young people with intellectual and developmental disabilities might be. However, the current research project recruited young people with intellectual and developmental disabilities from a particular type of special college in the Czech Republic; thus, the results may not be representative of young adults with intellectual and developmental disabilities in other post‐secondary educational contexts. For example, all of the young people with intellectual and developmental disabilities recruited had an intellectual disability, and some of them were also autistic. The focus groups did not include young people with developmental disabilities who did not have an intellectual disability. Those who were autistic without disabilities may have had different experiences. Similarly, young adults with intellectual and developmental disabilities who attended a special college for students with more severe disabilities and their parents and teachers might have a different view on transition from those in the specific setting included in this project. There was also little variation in characteristics within the teacher and other professional groups.

Although the sample size in terms of the number of focus groups and the number of participants overall was limited, the data from all the focus groups achieved saturation. In addition, as recruitment was on a completely voluntary basis, it is possible that self‐selection bias may be a factor.

Each focus group involved just one stakeholder group, which may have limited the opportunity for cross‐fertilisation of ideas and responses to the ideas of others. However, managing the group dynamics and, in particular, encouraging young people and parents to give their views in the presence of teachers and other professionals would potentially have been more challenging in more mixed groups.

Future research could usually allow a longer time frame for each group—in our study, the groups were limited to 60 min to minimise participant burden. However, future research could usefully allow more time to discuss potential barriers and facilitators and gather more detailed examples of transition successes and of what happens when transition support is not available or fails. More information about the nature and impact of transition support at school and in other settings will also be particularly useful.

### 8.3 Towards a Conceptual Model of Transition and a Framework for Future Research

Despite these limitations, the findings from this study provide new insights into how transition is conceptualised and what is important to different stakeholders. They also illustrate the need to connect the findings to broader theories.

Participants in the current study identified transition outcomes in all eight quality‐of‐life domains (Schalock et al. 2002) (see Table 3). The Quality‐of‐Life framework has generally been accepted as useful for conceptualising the quality (outcomes) of services—it is sometimes referred to as a “sensitising notion” for helping service providers to understand what they are aiming to provide. It has also been seen as useful as a measurement tool—both of outcomes of services but also more generally of whether the UN Convention on the Rights of People with Disabilities is being implemented (Gómez et al. 2020). Beadle‐Brown et al. (2023) suggested that it would be potentially useful to conceptualise the indicators of successful transition (i.e., the outcomes of the transition process) in terms of the eight quality of life domains originally set out in the international consensus on the quality of life and to use this to measure the quality and effectiveness of transition support services.

**TABLE 3:** Examples of indicators of each quality‐of‐life domain identified in the context of successful transition to adulthood.

Quality‐of‐life domain	Examples of indicators identified in the context of a successful transition
Social inclusion	Participating in community activities
	Feeling included
Self‐determination	Self‐determination and making decision
	Being responsible
Rights	Respect
	Freedom from abuse and discrimination
	Equal opportunities
Emotional well‐being	Strength and courage
	Happiness/Satisfaction
	Self‐esteem and confidence
Social relationships	Friendships and social networks
	Having a partner/personal relationship
	Having a family of one's own
Physical well‐being	Being safe
	Having physical needs met
Material well‐being	Income (from employment)
	Having own home
Personal development and meaningful occupation	Learning skills and becoming more independent.
	Personal growth
	Being active and participating—in work, leisure, etc.

Future research could use this framework at least as one of the lenses on successful transition, asking the question, do young persons with disabilities go on to experience a quality of life similar to those without disabilities during and post‐transition to adulthood? Do people receive the support they need in all aspects of their lives to achieve this?

### 8.4 Implications for Practice

Being included in society, increasing social networks, and positive relationships were highlighted as important elements of a successful transition. There was also a recognition that young people with intellectual and developmental disabilities were vulnerable to exclusion and being bullied or taken advantage of to a greater extent than young people without intellectual and developmental disabilities. However, there was little recognition in any of the focus groups of the impact of the special and segregated trajectory that young people with intellectual and developmental disabilities generally follow (Šiška et al. 2018). Although opportunities just like everyone else were a key theme, young people with intellectual and developmental disabilities have traditionally been denied these opportunities as a result of such a segregated pathway. More efforts are needed at different levels across the system to bring about an inclusive education system that supports learning and development and helps young people to build relationships and experience inclusion in the community more generally. This is not just an issue in the Czech Republic—segregated educational trajectories are still a reality for young people with intellectual and developmental disabilities in many other countries.

A lack of focus on supporting young people to learn independent living skills and job‐related skills at a younger age was also identified. This often only appeared to happen if their family had facilitated this. The need for a greater focus on transition preparation and planning for this group of young people was highlighted in the focus groups and as reported in other papers from the same study currently in preparation (blind for review), the need to start the process earlier and include more skill building elements has important implications for both policy and practice. Implementing person‐centred planning to capture young people's dreams and aspirations and help them and their Circles of Support to plan for their future based on their dreams and wishes, skills, and interests could be an important step in changing this situation. However, person‐centred planning on its own is not enough to ensure positive futures and good outcomes and widespread implementation of person‐centred planning has generally been found to be difficult, despite requirements in policy mandating the use of such planning (Mansell and Beadle‐Brown 2004; Taylor and Taylor 2013; Caldwell et al. 2023).

The need for young people to increase their experiences and skills in integrated settings in the community is likely to be key to a successful transition. This is not just relevant to employment as had been found in Australia (Bigby and De Losa 2021; Foley et al. 2013)—but to all elements of transition as conceptualised by participants in this study.

The availability of support throughout the process of transition (and indeed throughout life) was seen as critical for successfully becoming an adult, particularly for people with intellectual disabilities. However, for people to develop new experiences, be involved in new activities, and make informed choices, the support needs to enable and empower them, not control and do for them (Mansell and Beadle‐Brown 2012). Nevertheless, there were many barriers to this happening, for example, low expectations of what people with intellectual disabilities are capable of, lack of available support, lack of training for those providing support, and lack of cooperation and collaboration within and across different agencies and sectors—education, employment support, social services, housing—as well as partnership with the individual themselves and their Circle of Support. Processes and incentives for greater collaboration focused on the individual with intellectual and developmental disabilities and their quality of life are critical to improving the fragmentation caused by silo working. Again, well‐implemented person‐centred planning can help here, but getting different organisations to the table in the first place is often challenging (Šiška and Beadle‐Brown 2022). Future papers will explore the perceived barriers and facilitators of successful transition in more detail.

## Funding

This project was supported by the Czech Science Foundation, project GACR/PEDAL ID 22‐26896S, Cooperatio Programme, Charles University.

## Ethics Statement

All procedures performed in this study were in accordance with the ethical standards of the institutional research committee and with the 1964 Helsinki Declaration and its later amendments. Informed consent was obtained from all individual participants involved in the study.

## Conflicts of Interest

The authors declare no conflicts of interest.

## Data Availability

The data that support the findings of this study are available from the corresponding author.
